# Investigation of atovaquone-induced spatial changes in tumour hypoxia assessed by hypoxia PET/CT in non-small cell lung cancer patients

**DOI:** 10.1186/s13550-021-00871-x

**Published:** 2021-12-29

**Authors:** Pauline Bourigault, Michael Skwarski, Ruth E. Macpherson, Geoff S. Higgins, Daniel R. McGowan

**Affiliations:** 1 Department of Oncology, University of Oxford, Oxford, OX3 7DQ UK; 2 Department of Oncology, Oxford University Hospitals NHS Foundation Trust, Oxford, UK; 3 Department of Clinical Oncology, Guy’s and St Thomas’ NHS Foundation Trust, London, UK; 4 Department of Radiology, Oxford University Hospitals NHS Foundation Trust, Oxford, UK; 5 Department of Medical Physics and Clinical Engineering, Oxford University Hospitals NHS Foundation Trust, Oxford, UK

**Keywords:** Non-small cell lung cancer, Tumour hypoxia, Atovaquone, PET, FMISO

## Abstract

**Background:**

Tumour hypoxia promotes an aggressive tumour phenotype and enhances resistance to anticancer treatments. Following the recent observation that the mitochondrial inhibitor atovaquone increases tumour oxygenation in NSCLC, we sought to assess whether atovaquone affects tumour subregions differently depending on their level of hypoxia.

**Methods:**

Patients with resectable NSCLC participated in the ATOM trial (NCT02628080). Cohort 1 (*n* = 15) received atovaquone treatment, whilst cohort 2 (*n* = 15) did not. Hypoxia-related metrics, including change in mean tumour-to-blood ratio, tumour hypoxic volume, and fraction of hypoxic voxels, were assessed using hypoxia PET imaging. Tumours were divided into four subregions or distance categories: edge, outer, inner, and centre, using MATLAB.

**Results:**

Atovaquone-induced reduction in tumour hypoxia mostly occurred in the inner and outer tumour subregions, and to a lesser extent in the centre subregion. Atovaquone did not seem to act in the edge subregion, which was the only tumour subregion that was non-hypoxic at baseline. Notably, the most intensely hypoxic tumour voxels, and therefore the most radiobiologically resistant areas, were subject to the most pronounced decrease in hypoxia in the different subregions.

**Conclusions:**

This study provides insights into the action of atovaquone in tumour subregions that help to better understand its role as a novel tumour radiosensitiser.

*Trial registration*: ClinicalTrials.gov, NCT0262808. Registered 11th December 2015, https://clinicaltrials.gov/ct2/show/NCT02628080

**Supplementary Information:**

The online version contains supplementary material available at 10.1186/s13550-021-00871-x.

## Background

Lung cancer is the leading cause of cancer-related death in the UK with a five-year overall survival rate only in the region of 16% [1]. Non-small cell lung cancer constitutes 80% of lung cancer cases [2] and is associated with poor survival despite advances in the delivery of multi-modality treatment. Modern treatment of NSCLC is complex and increasingly involves utilisation of several treatment modalities, such as surgery, radiotherapy (RT), systemic therapies (chemotherapy, immunotherapy, and targeted agents), and interventional radiology and palliative care [3]. Modelling using multivariate analysis with Monte Carlo simulation reported that around 77% of patients with lung cancer have an evidence-based indication for RT during their course of disease [4]. Many solid tumours, including NSCLC, are typically dependent upon an abnormal, poorly functioning vasculature for oxygen delivery [5]. Coupled with the high metabolic requirements of many tumours, this leads to an imbalance in oxygen supply and demand and so causes tumour hypoxia. Importantly, various pre-clinical and clinical studies have highlighted that tumour hypoxia enhances resistance to anticancer treatments, particularly RT, and promotes an aggressive tumour phenotype [6].

Oxygen deprivation or hypoxia is defined as an oxygen tension under physiological normoxia (less than or equal to 2.03–3.04 kPa) [7]. Hypoxia is closely associated with several ‘hallmarks of cancer’ such as reprogramming energy metabolism, inducing angiogenesis, and resisting cell death [8]. Gray and colleagues suggested for the first time in the 1950s that hypoxia could influence radiotherapy outcomes as the radioresistance of hypoxic tumour cells increases by a factor of up to three compared to normoxic tumour cells [5, 9, 10].

To reduce tumour hypoxia and therefore enhance radiosensitivity, decreasing cellular oxygen consumption rate (OCR) via metabolic reprogramming has arisen as a promising approach [11]. Mathematical modelling indicated that a 30% reduction in OCR would abrogate severe tumour hypoxia and could stand as a more efficient strategy than enhancing blood oxygen levels or augmenting blood flow [12].

Notably, the well-tolerated anti-malarial drug atovaquone has been shown to decrease OCR in multiple cancer cell lines, alleviate hypoxia in spheroids and xenografted tumours, and induce radiosensitisation [13]. Atovaquone acts as a mitochondrial inhibitor of oxidative phosphorylation at complex III (the cytochrome *bc*_1_ complex) of the electron transport chain (ETC) [14]. Moreover, in a translational clinical trial, we recently assessed, through the analysis of gene expression and pharmacodynamics endpoints of tumour hypoxia, the clinical potential of atovaquone as a mitochondrial inhibitor in patients with NSCLC [15]. This study reported for the first time that a mitochondrial inhibitor can be employed to target mitochondrial metabolism and modify the tumour microenvironment through hypoxia reduction in patients so as to potentially improve the efficacy of radiotherapy [15].

Following the observation that atovaquone results in hypoxia reduction in NSCLC patients [15], we hypothesised that atovaquone may affect tumour subregions differently depending on their level of hypoxia. Thus, the aim of the present study was to investigate atovaquone-induced changes in hypoxia in tumour subregions by analysing hypoxia PET-CT scans.

## Methods

### Patients

Patients with resectable NSCLC were recruited for the open-label, non-randomised, equal-sized two-cohort ATOM clinical trial (NCT02628080) completed at the Oxford Cancer and Haematology Centre (UK) in accordance with the provisions of the Declaration of Helsinki and Good Clinical Practice guidelines. Patients in cohort 1 (*n* = 15) received oral atovaquone (Wellvone, 750 mg/5 mL micronised suspension, GlaxoSmithKline) twice daily. Patients were asked to take atovaquone orally together with fat-containing food to aid absorption. Patients in cohort 2 (*n* = 15) did not receive atovaquone. Eligible patients were aged ≥ 18 years, had a pathologic or radiological diagnosis of NSCLC, were scheduled for surgical resection, had disease > 2 cm in diameter, and had Eastern Cooperative Oncology Group (ECOG) performance status 0–2. Patients were excluded if taking known ETC inhibitors. Despite a male predominance in untreated patients, the main clinical characteristics were well balanced in the two cohorts. The clinical characteristics of patients are shown in Table 1. For full details regarding trial design, patients, and treatment, the reader is referred to [15].

**Table 1:** Clinical characteristics of patients

Patient ID	Cohort	Atovaquone	Interval between scans (days)	PET tracer	Age (years)/sex	TNM staging	Tumour volume (mL)
10001	1	Yes	13	FMISO	58/M	T3 N0 M0	89.5
10004	1	Yes	8	FAZA	71/F	T4 N3 M0	184.9
10005	1	Yes	14	FMISO	72/M	T2a N2 M0	61.0
10,006	1	Yes	14	FMISO	69/M	T4 N2 M0	179.5
10008	1	Yes	10	FMISO	77/F	T2a N0 M0	17.8
10009	1	Yes	14	FMISO	78/M	T3 N0 M0	59.3
10010	1	Yes	13	FMISO	70/M	T4 N2 M0	371.1
10011	1	Yes	8	FMISO	77/F	T4 N0 M0	24.6
10013	1	Yes	9	FMISO	54/F	T1c N3 M0	15.5
10014	1	Yes	13	FMISO	55/F	T3 N0 M0	42.2
10016	1	Yes	14	FMISO	57/F	T2a N0 M0	30.6
10019	1	Yes	14	FMISO	58/M	T4 N0 M0	366.9
10020	1	Yes	14	FMISO	73/M	T3 N0 M0	85.3
10022	1	Yes	14	FMISO	65/F	T2b N1 M0	45.9
10023	1	Yes	8	FMISO	58/F	T3 N0 M0	33.4
10024	2	No	13	FMISO	68/M	T3 N1 M0	45.8
10025	2	No	14	FMISO	71/M	T4 N0 M0	40.8
10028	2	No	14	FMISO	53/M	T3 N1 M0	20.6
10029	2	No	14	FMISO	87/M	T2b N1 M0	24.1
10030	2	No	7	FMISO	57/M	T3 N2 M0	243.0
10031	2	No	15	FMISO	58/F	T3 N0 M1b	5.4
10033	2	No	14	FMISO	75/M	T1c N0 M0	6.3
10034	2	No	14	FMISO	63/M	T1b N1 M0	43.7
10037	2	No	11	FMISO	70/M	T2a N0 M0	19.1
10039	2	No	14	FMISO	67/M	T4 N0 M0	144.8
10041	2	No	7	FMISO	61/M	T2a N1 M0	20.0
10043	2	No	7	FMISO	69/F	T4 N0 M0	80.7
10044	2	No	8	FMISO	62/F	T3 N1 M0	45.3
10045	2	No	2	FMISO	81/M	T3 N0 M0	185.4
10046	2	No	14	FMISO	70/M	T3 N0 M0	57.5

### Data acquisition

Change in tumour hypoxic volume (HV) was the primary imaging endpoint measured with hypoxia PET-CT. Single bed position image acquisition centred on the tumour was performed with GE Discovery 690 or 710 PET-CT Scanners (GE Healthcare) for 10 min at 4 hours following the administration of 18F-fluoromisonidazole (FMISO, 29 patients) (University of Cambridge) or 18F-fluoroazomycin-arabinofuranoside (FAZA, 1 patient) (University of Manchester) with an activity of 370 MBq. The same scanner and tracer were used for the two visits of each patient with baseline scans (indicated as ‘pre’) and pre-surgery scans (indicated as ‘post’) for atovaquone-treated and untreated patients. CT images provided attenuation correction and localisation. Patients in cohort 1 had a median length of 13 (IQR, 9–14) days between imaging time points, depending on their planned date for surgery. Patients in cohort 2 had a median length of 14 (IQR 7–14) days between imaging time points.

### Image analysis

Tumours on four-hour hypoxia PET-CT images were manually outlined on the CT image by an experienced radiologist and copied to the co-registered PET image. Images were analysed using Hermes Hybrid Viewer Software (Hermes Medical Solutions AB) and MATLAB (version R2021a, MathWorks, Natick, MA, USA). A matrix containing each voxel coordinates (*x*, *y*, *z*) along with the respective radiotracer standardised uptake value (SUV) per voxel was first extracted for every outlined tumour volume.

The background mean SUV (SUV_mean_) was obtained by outlining blood in the central portion of the descending aorta. To measure the HV, each tumour voxel’s SUV was divided by the background SUV_mean_ to determine the tumour-to-blood ratio (TBR) value per voxel:

1 $${\text{TBR = }}\frac{{{\text{SUVvoxel}}}}{{\text{SUVmean descending aorta}}}$$

As described by Koh et al. [16], voxels with a TBR equal to or greater than 1.4 were classified as hypoxic. Volumes of hypoxic voxels and TBR_mean_ values were compared pre- versus post-atovaquone. A decrease in HV equal to or greater than 10% from baseline was described as a meaningful decrease after atovaquone treatment, in accordance with a previous clinical study [17]. The minimum detectable change (MDC) method and FMISO test–retest reproducibility data were used to determine this cut-off [18]. For additional explanation regarding this threshold, the reader is referred to [15], Supplementary Materials and Methods.

Changes in hypoxia following atovaquone treatment were studied in tumours overall and in tumour subregions. In-house MATLAB (version R2021a, MathWorks, Natick, MA, USA) code was first used to calculate the distance of every tumour voxel to the nearest edge of the outlined tumour. Voxel dimensions on PET-CT images were 2.7 × 2.7 × 3.3 mm^3^. Voxels were then divided into four subregions or distance categories: *edge* (the outermost shell of voxels), *outer* (voxels’ centre up to 5.5 mm of the tumour outline), *inner* (voxels’ centre between 5.5 and 11 mm of the tumour outline), and *central* (voxels’ centre superior to 11 mm inside the tumour outline), in line with a previous PET study about FMISO uptake in advanced NSCLC [19].

### Statistical analysis

Statistical analyses used IBM SPSS Statistics (version 27). The normality of data was inspected using Shapiro–Wilk test. For each distance category, the chi-square test of homogeneity was used to assess the significance of pre- to post-atovaquone changes in the proportions of voxels assigned to each region. Percentage change in TBR_mean_ and HV was calculated between trial visits for each patient. The binomial method was used to derive 95% confidence intervals (CI) for median changes within cohorts. The statistical significance of the difference of means and medians of TBR_mean_ and HV in each distance category pre- to post-atovaquone was evaluated using the Welch’s *t* test for samples of unequal variance. For multiple comparisons in *t* tests, a Bonferroni-corrected *P* value < 0.05 was considered significant.

## Results

### Changes of TBR in tumour subregions

**Fig. 1:**
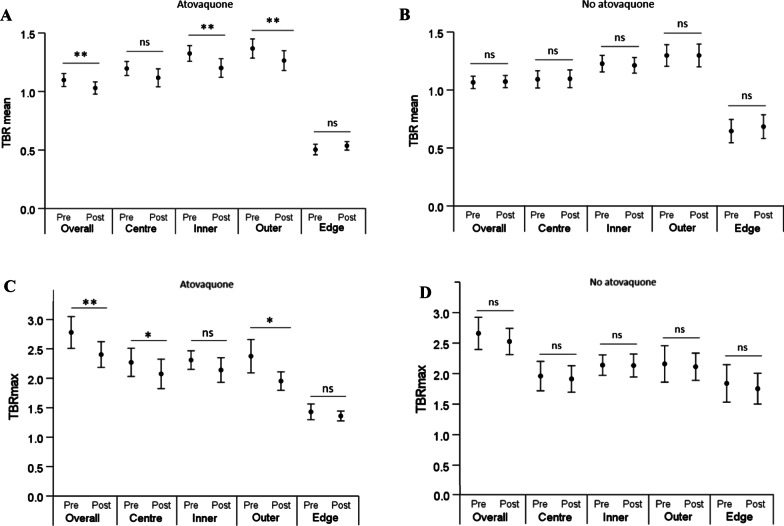
TBR_mean_ scores in tumours overall and tumour subregions at baseline scans (indicated as ‘pre’) and pre-surgery scans (indicated as ‘post’) for atovaquone-treated (**A**) and untreated (**B**) patients. TBR_max_ scores in tumours overall and tumour subregions at ‘pre’ and ‘post’ for atovaquone-treated (**C**) and untreated (**D**) patients. Results indicated as mean ± standard error. *, *P* < 0.05; **, *P* < 0.01; ns, non-significant

Atovaquone-induced changes in hypoxia were initially assessed in tumours overall and in tumour subregions, using four-hour hypoxia PET-CT. TBR_mean_ was the first hypoxia-related score evaluated. TBR_mean_ decreased significantly from baseline in atovaquone-treated patients in tumours overall (1.11 to 1.01, *P* < 0.01) as well as in the inner (1.32 to 1.20, *P* = 0.01) and outer (1.37 to 1.26, *P* < 0.01) subregions (Fig. 1A). However, there was no significant reduction in TBR_mean_ from baseline in the centre subregion (1.19 to 1.12, *P* = 0.07). Notably, baseline mean TBR_mean_ in the inner [1.32 (95% CI 1.2–1.5)] and outer [1.37 (95% CI 1.2–1.5)] subregions exceeded baseline mean TBR_mean_ in the centre subregion [1.19 (95% CI 1.0–1.3)]. Change in mean TBR_mean_ in the non-hypoxic edge subregion following treatment was negligible [0.50 (95% CI 0.4–0.6) to 0.54 (95% CI 0.5–0.6), *P* = 0.23]. In contrast, no significant change was observed in TBR_mean_ in untreated patients, neither in tumours overall nor in any tumour subregion (Fig. 1B).

The overall change in TBR_mean_ from baseline was − 9.0% in atovaquone-treated patients against + 3.7% in untreated patients. In atovaquone-treated patients, change in TBR_mean_ from baseline was − 5.8% in centre, − 9.1% in inner, and − 8.0% in outer subregions. In untreated patients, negligible change in TBR_mean_ was observed in these three subregions (0.9%, − 1.6%, and 0.0%, respectively). TBR_mean_ increased from baseline in the edge region in both cohorts similarly, by + 8.0% in atovaquone-treated and by + 4.6% in untreated patients. Thus, atovaquone caused a decrease in TBR_mean_ in tumours overall and in all tumour subregions except the edge region. Interestingly, the reduction in TBR_mean_ in the centre subregion was lower than in the inner and outer subregions. TBR_max_ values showed a similar trend with no significant changes from baseline in each distance category and overall for untreated patients (Fig. 1D) but was significantly reduced in two distance categories as well as overall for treated patients (Fig. 1C).

### Changes of tumour hypoxic volume

**Fig. 2:**
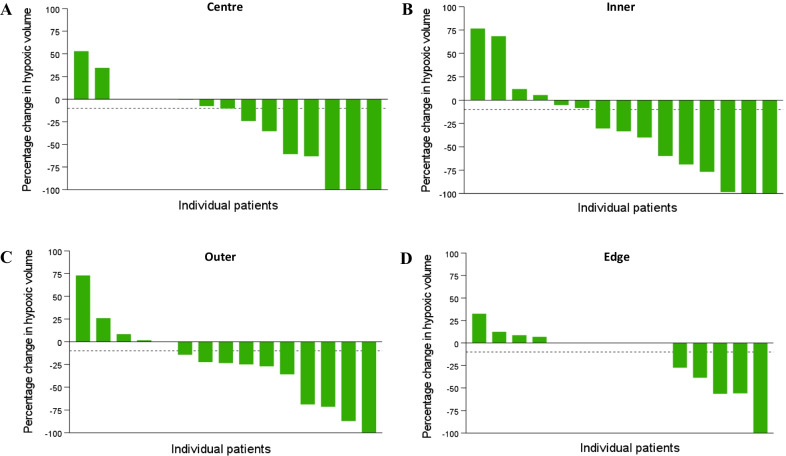
Change in tumour HV measured by hypoxia PET-CT. Waterfall plots of percentage change in HV in tumour subregions for atovaquone-treated patients: centre (**A**), inner (**B**), outer (**C**), and edge (**D**). TBR ≥ 1.4 is used to define HV. A reduction in HV ≥ 10% was considered meaningful (dashed line). All atovaquone-treated patients including those with no change in HV between baseline scan and pre-surgery scan are included. Waterfall plots of percentage change in HV in tumour subregions for untreated patients are presented in Supplementary Fig. 1

Atovaquone-induced changes in tumour HV were then assessed. TBR equal to or greater than 1.4 was first used to define HV. Eleven (73.3%) atovaquone-treated patients had an overall and meaningful (equal to or greater than 10%) decrease in HV from baseline, and the median change was − 28% (95% CI − 58.2 to − 4.4). Meanwhile, only two (13.3%) untreated patients had an overall reduction in HV equal to or greater than 10%, and the median change was + 15.5% (95% CI − 6.5 to 35.5), as previously reported [15]. Following atovaquone treatment, eight patients (53%) also had a meaningful decrease in HV in the centre subregion, with a median change of − 10.4% (95% CI − 54.2 to − 1.05) (Fig. 2A). Nine (60%) patients had a meaningful reduction in HV in the inner subregion, which had the greatest median change of − 33.3% (95% CI − 61.7 to 0.6), as well as in the outer subregion, with a median change of − 23.5% (95% CI − 49.4 to 0.5) (Fig. 2B, C). Lastly, five (33%) patients had a meaningful reduction in HV in the edge subregion, even if the median change was 0.0% (95% CI − 33.7 to 4.6) (Fig. 2D). In contrast, median change in untreated patients was + 11.5% (95% CI − 2.8 to 32.1) in the outer subregion, + 1.5% (95% CI − 7.4 to 7.3) in the inner subregion, and 0.0% in the centre (95% CI − 2.1 to 3.6) and edge (95% CI − 5.1 to 5.7) subregions. A meaningful reduction in HV was observed in only 2 (13.3%) untreated patients in the inner, outer, and edge subregions (Additional file 1: Figure S1).

In both cohorts, the magnitude of tumour HV at baseline was not in itself predictive of hypoxia reduction to atovaquone. For instance, the two untreated patients that displayed a meaningful reduction in HV in tumours overall had either medium (44.8 mL) or very small (3.5 mL) baseline HV. Similarly, no meaningful decrease in HV was observed in four atovaquone-treated patients, who had large (366.9 mL), medium (45.9 and 59.3 mL), and small (24.6 mL) baseline HVs.

As median HV decreased from baseline in the centre, inner, and outer tumour subregions, analysis of more intense tumour hypoxia was performed. TBR greater than 1.6 and 1.8 were used to define tumour hypoxic voxels. Only tumours that had sufficient HV (predefined as ≥ 1.5 mL) at these TBR thresholds to accurately assess change were included. Using TBR greater than 1.6 and 1.8, atovaquone-induced reduction in HV was even more pronounced in all tumour subregions except in the edge subregion where no median change in HV was consistently observed (Table 2).

**Table 2:** Summary of tumour hypoxic volume results using 4-h hypoxia PET-CT. Three thresholds were used to define progressively higher hypoxia PET tracer uptake: TBR ≥ 1.4 (a), TBR ≥ 1.6 (b), and TBR ≥ 1.8 (c)

	Atovaquone cohort (*n* = 15)				No atovaquone cohort (*n* = 15)			
	Centre	Inner	Outer	Edge	Centre	Inner	Outer	Edge
(a)								
Baseline scan	15.2 (3.1–39.7)	29.8 (5.7–44.6)	18.1 (5.9–43.8)	0.0 (0.0–2.9)	2.2 (0.0–28.5)	6.0 (2.2–26.6)	7.3 (2.1–23.9)	0.0 (0.0–8.1)
Pre-surgery scan	9.2 (0.0–32.1)	17.9 (1.5–23.4)	10.1 (1.9–27.2)	1.44 (0.0–3.4)	2.1 (0.0–32.1)	6.8 (2.3–23.4)	9.6 (2.3–27.2)	0.5 (0.0–11.4)
Change from baseline	− 10.4% (− 54.2 to − 1.05)	− 33.3% (− 61.7 to 0.6)	− 23.5% (− 49.4 to 0.5)	0.0% (− 33.7 to 4.6)	0.0% (− 5.6 to 9.6)	+ 0.2% (− 9.1 to 22.2)	+ 20.9% (− 0.4 to 29.8)	0.0% (− 13.7 to 7.0)

	Atovaquone cohort (*n* = 12)				No atovaquone cohort (*n* = 11)			
	Centre	Inner	Outer	Edge	Centre	Inner	Outer	Edge
(b)								
Baseline scan	9.3 (0.0–16.8)	10.2 (2.4–34.8)	20.8 (8.9–57.4)	0.0 (0.0–1.9)	2.4 (0.0–15.8)	2.5 (0.2–19.2)	6.1 (9.5–18.3)	0.0 (0.0–5.4)
Pre-surgery scan	3.8 (0.0–17.6)	4.7 (0.3–29.7)	3.8 (0.6–32.1)	0.0 (0.0–2.4)	0.5 (0.0–13.0)	3.1 (0.6–15.4)	6.9 (1.0–19.1)	0.0 (0.0–5.2)
Change from baseline	− 11.3% (− 45.5 to 2.0)	− 49.3% (− 66.9 to − 4.4)	− 50.5% (− 61.6 to 2.1)	0.0% (− 28.9 to 29.6)	0.0% (− 11.3 to 8.5)	0.0% (− 18.2 to 28.8)	+ 5.1% (− 10.3 to 34.8)	0.0% (− 8.5 to 8.0)

	Atovaquone cohort (*n* = 9)				No atovaquone cohort (*n* = 8)			
	Centre	Inner	Outer	Edge	Centre	Inner	Outer	Edge
(c)								
Baseline scan	3.5 (0.0–11.7)	2.0 (0.5–22.8)	4.0 (0.4–22.9)	0.0 (0.0–0.7)	0.0 (0.0–7.9)	1.4 (0.0–7.9)	0.5 (0.0–10.9)	0.0 (0.0–3.7)
Pre-surgery scan	0.7 (0.0–11.2)	0.4 (0.0–14.6)	0.9 (0.0–13.3)	0.0 (0.0–0.0)	0.0 (0.0–6.5)	1.3 (0.1–7.6)	3.4 (0.3–10.3)	0.0 (0.0–6.5)
Change from baseline	− 14.8% (− 46.9 to 5.42)	− 23.7% (− 65.9 to 6.6)	− 81.0% (− 84.6 to − 5.6)	0.0% (− 41.6 to 24.3)	0.0% (− 19.6 to 8.7)	0.0% (− 23.8 to 10.5)	+ 6.0% (− 18.8 to 12.8)	0.0% (− 7.2 to 7.4)

Median values (mL) with IQR for baseline and pre-surgery scans, and median percentage change from baseline with 95% CI

Given that the treatment duration differed between patients, its influence on tumour HV changes was evaluated (Additional file 1: Figure S2). The three patients with the greatest reduction in HV from baseline (− 100%, − 86% and − 69%) received atovaquone for more than 11 days. However, the only two patients who had an increase in HV from baseline were also in this group. Overall, no relationship was observed between changes in HV from baseline and treatment time. There was a high correlation between the hypoxic volume (based on TBR > 1.4) and total tumour volume (examined for pre-only patients). The correlation decreased for higher TBR thresholds. This is shown in Additional file 1: Figure S3.

### Changes in the fraction of tumour hypoxic voxels

**Fig. 3:**
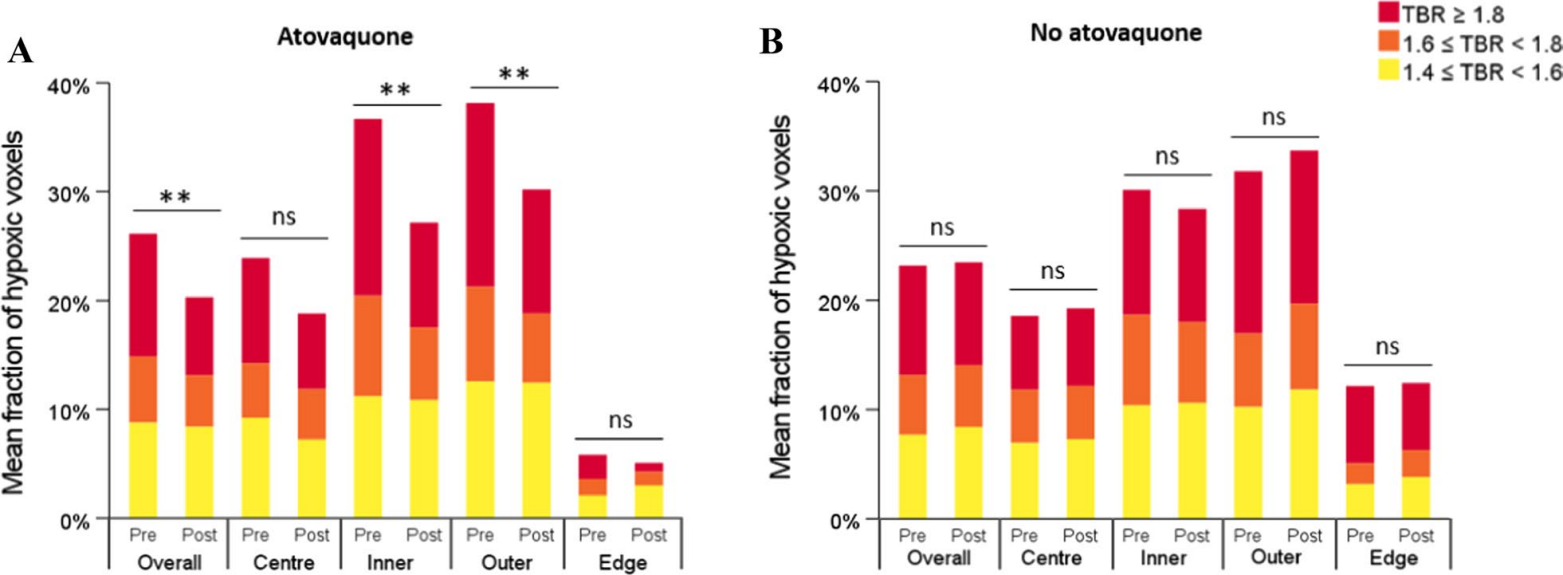
Changes in the fraction of hypoxic voxels from baseline in atovaquone-treated (**A**) and untreated (**B**) patients. Tumour hypoxic voxels are divided into three groups (yellow, orange, red) according to their level of hypoxia defined by their TBR value. Statistical significances are only indicated for global changes in the mean fraction of hypoxic voxels between visits (i.e. baseline visit and pre-surgery visit named ‘pre’ and ‘post’, respectively) not all three TBR thresholds. **, *P* < 0.01; ns, non-significant

Following the analysis of atovaquone-induced changes in HV in tumour subregions using different TBR thresholds to define hypoxia, variations in the fraction of tumour hypoxic voxels were investigated (Fig. 3). Using TBR ≥ 1.4 to define hypoxia, globally, the mean fraction of hypoxic voxels decreased significantly from baseline in atovaquone-treated patients (*P* < 0.001) contrary to untreated patients where no significant change was observed (*P* = 0.63) (Table 3).

**Table 3:** Summary of the fraction of hypoxic voxels at baseline (‘pre’) and pre-surgery (‘post’) visits for atovaquone-treated (a) and untreated (b) patients overall and in tumour subregions, using four-hour hypoxia PET-CT analysis. Voxels with a TBR ≥ 1.4 were defined as hypoxic. Mean values and IQR are shown for pre and post with *P* values for the difference pre to post

	Overall		Centre		Inner		Outer		Edge	
	Pre	Post	Pre	Post	Pre	Post	Pre	Post	Pre	Post
(a)										
Mean	26.1%	20.3%	23.9%	18.8%	36.7%	27.2%	38.2%	30.2%	5.8%	5.1%
IQR	4.4–45.8%	0.0–33.5%	7.7–37.1%	0.0–35.4%	17.3–60.4%	4.8–44.2%	14.3–57.1%	11.4–42.3%	0.0–8.2%	0.0–7.6%
* P*	< 0.001		0.05		0.01		0.01		0.86	
(b)										
Mean	23.2%	23.0%	6.2%	6.4%	10.0%	9.5%	10.6%	11.2%	4.1%	4.2%
IQR	0.0–43.9%	0.0–42.1%	0.0–35.5%	0.0–35.6%	8.0–51.4%	9.1–44.5%	13.9–54.4%	20.4–49.0%	0.0–14.61%	0.0–24.5%
* P*	0.63		0.72		0.47		0.53		0.82	

In particular, with treatment, a significant reduction from baseline in the mean fraction of hypoxic voxels was identified in the inner (*P* = 0.01) and outer (*P* = 0.01) subregions. The reduction was non-significant in the centre (*P* = 0.05) and negligible in the edge subregion (*P* = 0.86) (Table 3a). In untreated patients, despite a trend towards an increase from baseline in the mean fraction of hypoxic voxels in the centre, outer, and edge subregions, or reduction in the inner subregion, no change was statistically significant (Table 3b).

Different TBR thresholds were then used to compare changes in the fraction of progressively more hypoxic voxels (Fig. 3A, B). Regarding atovaquone-treated patients, in the 1.4 ≤ TBR < 1.6 category, there was no significant change from baseline in the mean fraction of voxels neither overall (*P* = 0.96) nor in centre (*P* = 0.31), inner (*P* = 0.95), outer (*P* = 0.91), and edge (*P* = 0.21) subregions (Table 4a). In the 1.6 ≤ TBR < 1.8 category, a significant reduction from baseline was only observed in tumours overall (*P* = 0.01) (Table 4b). In the TBR ≥ 1.8 category, a significant reduction from baseline was observed in tumours overall (*P* < 0.001) and in the inner (*P* < 0.01) and outer (*P* = 0.01) subregions (Table 4c). In untreated patients, there was no significant change from baseline in the mean fraction of voxels in any TBR category, neither overall in tumours nor in subregions.

**Table 4:** Summary of the fraction of hypoxic voxels at baseline (‘pre’) and pre-surgery (‘post’) visits for atovaquone-treated patients, with four-hour hypoxia PET-CT. Three TBR categories were used to define progressively higher hypoxia PET tracer uptake: 1.4 ≤ TBR < 1.6 (**a**), 1.6 ≤ TBR < 1.8 (**b**), and TBR ≥ 1.8 (**c**). Mean values and IQR are shown for pre and post with *P* values for the difference pre to post

	Overall		Centre		Inner		Outer		Edge	
	Pre	Post	Pre	Post	Pre	Post	Pre	Post	Pre	Post
(a)										
Mean	8.8%	6.8%	9.2%	7.2%	11.2%	10.9%	12.6%	12.5%	2.1%	2.9%
IQR	3.8–12.4%	0.0–13.9%	6.1–13.4%	0.0–12.0%	8.6–13.7%	4.2–17.1%	9.2–15.4%	8.0–17.0%	0.0–3.4%	0.0–5.4%
* P*	0.96		0.31		0.95		0.91		0.21	
(b)										
Mean	6.1%	4.7%	5.0%	4.7%	9.2%	6.7%	8.7%	6.4%	1.5%	1.3%
IQR	0.0–9.5%	0.0–8.6%	0.0–6.9%	0.0–7.9%	5.1–12.8%	0.7–10.4%	3.0–12.1%	1.5–11.8%	0.0–1.5%	0.0–2.3%
* P*	0.01		0.72		0.96		0.13		0.60	
(c)										
Mean	11.3%	7.2%	9.6%	6.9%	16.3%	9.6%	16.9%	11.4%	2.2%	0.8%
IQR	0.0–19.3%	0.0–4.6%	0.0–14.5%	0.0–7.7%	0.9–31.0%	0.0–8.0%	1.2–26.8%	0.0–7.7%	0.0–2.1%	0.0–0.0%
* P*	< 0.001		0.11		0.009		0.01		0.27	

Thus, it seems that atovaquone did decrease the global fraction of hypoxic voxels significantly in tumours overall and in all subregions except the edge subregion. The fraction of the most intensely hypoxic voxels also showed the greatest reduction among tumour hypoxic voxels and in most tumour subregions.

## Discussion

Following the recent demonstration that the mitochondrial inhibitor atovaquone decreases tumour hypoxia in patients with NSCLC, this study shows that the reduction in TBR and HV occurred in the outer, inner, and centre tumour subregions. The centre subregion displayed a lower TBR at baseline which is likely to be due to lack of hypoxic PET tracer uptake due to a lack of viable tumour cells (e.g. necrotic regions) and may explain the lesser reduction observed in these regions. Besides, atovaquone did not seem to act at the edge subregion of the tumour. Baseline TBR_mean_ in this subregion was markedly lower than the hypoxia threshold set at 1.4, and although TBR_mean_ did increase during treatment, it was insufficient to classify these voxels as hypoxic, thus explaining the absence of change in median HV in the edge tumour subregion.

The increase in median HV and TBR_mean_ from baseline in tumours of untreated patients, signifying that tumour hypoxia intensified over time, reinforced the reduction observed in atovaquone-treated patients. Moreover, as the nitroimidazole-based tracers FMISO and FAZA used accumulate exclusively in viable cells at oxygen levels responsible for hypoxia-related radioresistance [20], any decrease in uptake should therefore result in an improvement in tumour response to radiation. Indeed, several clinical studies reported that high uptake of these tracers acts as a negative prognostic biomarker for patients undergoing RT [21, 22]. Notably, we observed that the most intensely hypoxic tumour voxels (with a TBR greater than 1.6 or 1.8), defining radiobiologically highly resistant regions, were subject to the most pronounced decrease in hypoxia in tumour subregions. This provides insights into the action of atovaquone as a novel tumour radiosensitiser.

As hypoxia appeared to decrease to a greater extent in the inner and outer tumour subregions than in the centre subregion in atovaquone-treated patients, we hypothesise that blood perfusion may impact the reported treatment outcome. TBR indices at 4 hours post-injection seemed to fall towards tumour centres, and it is possible that TBR could underestimate the extent of hypoxia, especially at tumour centres. These voxels could be poorly perfused (or potentially necrotic), leading to very low rates of FMISO influx which would cause TBR values to remain relatively low despite generating hypoxic conditions which would cause long-term tracer accumulation. Thus, it would be interesting to assess the relationship between tumour blood perfusion and TBR using baseline perfusion CT and hypoxia PET scans.

Fixed distance categories were used to assign voxels to a tumour subregion, in accordance with a previous study on FMISO PET kinetics in NSCLC [19]. Yet, tumour volumes varied, and for large tumours, a predominance of voxels was assigned to the centre subregion. We tested dividing tumours into five subregions instead of four, but no significant change of TBR in response to atovaquone between each subregion was observed. Alternatively, a method that determines distance categories based on the tumour volume could be employed. By ranking from lowest to highest the voxels’ minimum distance from the tumour outline, and then assigning 25% of the voxels with the lowest distance from the outline in the edge subregion, the next 25% of the voxels in the outer subregion, the next 25% of the voxels in the inner subregion, and the voxels left in the centre subregion, the voxels may be divided into the subregions more proportionally. This method was tried for the first three patients, but again, no significant change in TBR per subregion was observed in response to atovaquone.

Moreover, the resolution of a PET scanner is around 3–4 mm [23], whilst the molecular effect of oxygen ranges from nm to μm [24]. The width of PET image voxels is thus of this length or a little bit smaller, which is notably greater than that of an average hypoxic tumour region, causing PET image voxels to likely include both hypoxic and normoxic regions. This causes relatively low SUV values on PET scans using the radiotracer FMISO compared to FDG, whose uptake within individual voxels is more uniform [25]. Moreover, as the binding function of FMISO is nonlinear, and smooth instead of a simple threshold, several scenarios can engender the same SUV_mean_ in a voxel. As shown by Grimes et al (figure 5, [24]), one would expect roughly the same PET signal whether the entire voxel was at 4.2 mmHg, or the voxel was split in half between oxic (50%) and 1.4 mmHg (50%), or if a voxel was 25% anoxic (and viable) and 75% well oxygenated. Concerns about the interpretation of PET results could thus be raised as the radiobiological response would presumably vary between these scenarios, given the typical oxygen enhancement ratio curve.

Our analysis of hypoxia PET scans shows differences in hypoxia in different NSCLC tumour regions at baseline, which could be useful for RT dose guidance. Hypoxia-based RT dose painting is an appealing approach for cancer therapy [26, 27]. Additional RT is dosimetrically and spatially adapted in dose painting, using tumour response maps obtained by repeat imaging during RT [28]. FMISO PET has previously been investigated for dose painting in head and neck cancer [29]. As retention of hypoxia PET radiotracer exclusively happens within viable cells retaining a functional ETC, tumour subvolumes that are very hypoxic but necrotic will plausibly not receive unnecessary dose escalation. Higher RT dose is, however, likely to only benefit hypoxic cancer cells that display clonogenic replicative potential [26].

Hypoxia PET image repeatability has been demonstrated to be high with modern PET/CT systems using FMISO head and neck cancer (HNC) [30] and NSCLC [31] over 1–2 days and using 18F-EF5 over a median 7-day period [32] Whilst the ATOM study did not formally test the reproducibility of hypoxia PET imaging, within the untreated cohort there were no significant differences between *TBR*_mean_ between the two scans for the overall tumour or individual tumour regions.

## Conclusions

In this study, we provide insights into the action of atovaquone in different subregions of NSCLC tumours. Following the demonstration that atovaquone reduces tumour hypoxia in patients with NSCLC, we show that this reduction mostly occurs in the inner and outer tumour subregions, and to a lesser extent in the centre subregion. Importantly, the most intensely hypoxic tumour voxels, and therefore the most radiobiologically resistant areas, are subject to the most pronounced decrease in hypoxia. This enables a better understanding of the role of atovaquone as a novel tumour radiosensitiser. Further investigation is required to assess whether the PET radiotracer may underestimate the level of hypoxia in the innermost tumour subregion and whether perfusion is perhaps responsible, at least in part, for this observation.

## Supplementary Information

**Additional file 1: Supplementary Figure 1.** Change in tumour HV measured by hypoxia PET-CT. **Supplementary Figure 2**. Influence of treatment time on tumour hypoxic volume changes. **Supplementary Figure 3.** Relationship between baseline tumour hypoxic volume and total baseline tumour volume.

## Data Availability

Data are available under reasonable request to the corresponding author.

## Abbreviations

CI: Confidence interval
DNA: Deoxyribonucleic acid
ETC: Electron transport chain
^18^F: Fluorine-18
FAZA: 18F-fluoroazomycinarabinofuranoside
FDG: 18F-fluorodeoxyglucose
FMISO: 18F-fluoromisonidazole
HV: Hypoxic volume
IQR: Interquartile range
MDC: Minimum detectable change
NSCLC: Non-small cell lung cancer
OCR: Oxygen consumption rate
PET: Positron emission tomography
RT: Radiotherapy
SCLC: Small cell lung cancer
SUV: Standardised uptake value
SUV_mean_: Mean SUV
TBR: Tumour-to-blood ratio
TBR_mean_: Mean TBR
