# Supplementary material for: Investigation of atovaquone-induced spatial changes in tumour hypoxia assessed by hypoxia PET/CT in non-small cell lung cancer patients
Source: EJNMMI Res. 2021 Dec 29;11:130. doi: 10.1186/s13550-021-00871-x (PMC8716680; doi:10.1186/s13550-021-00871-x)
Supplement: Supplementary file 1 — Additional file 1: Supplementary Figure 1. Change in tumour HV measured by hypoxia PET-CT. Supplementary Figure 2. Influence of treatment time on tumour hypoxic volume changes. Supplementary Figure 3. Relationship between baseline tumour hypoxic volume and total baseline tumour volume. [file 13550_2021_871_MOESM1_ESM.docx]

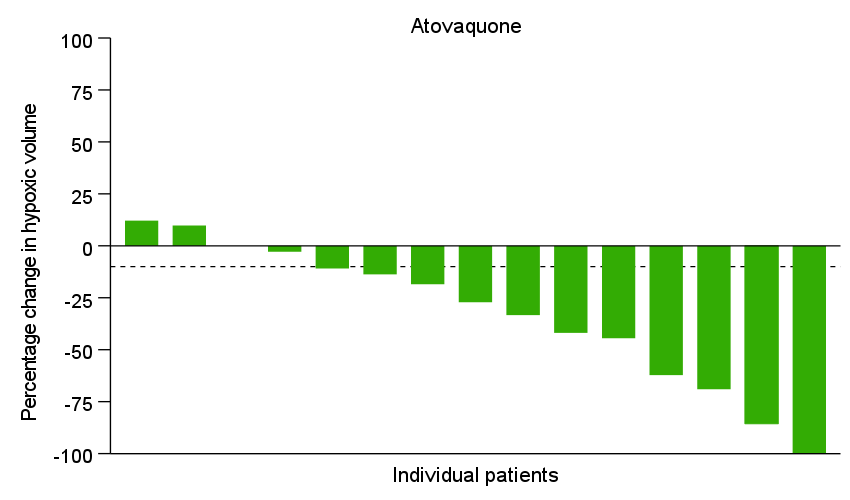

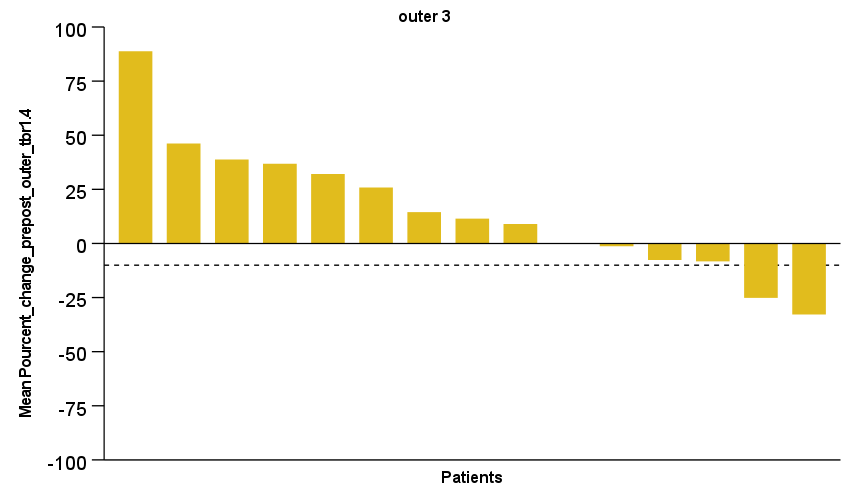

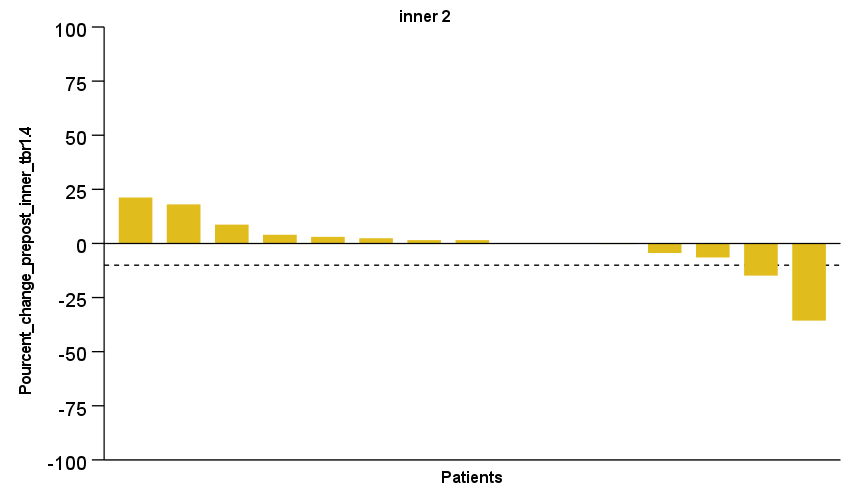

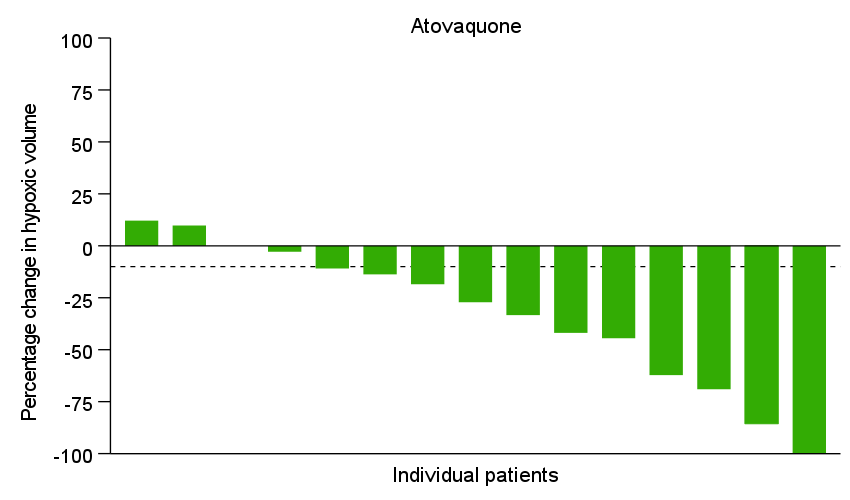

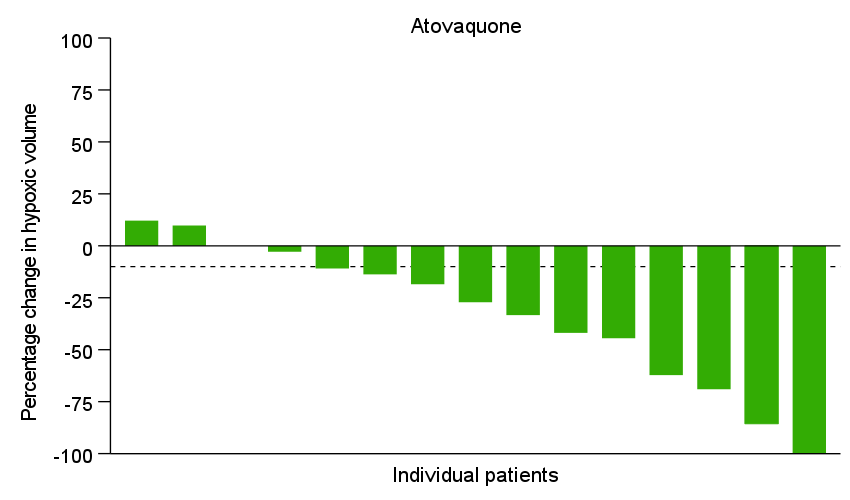

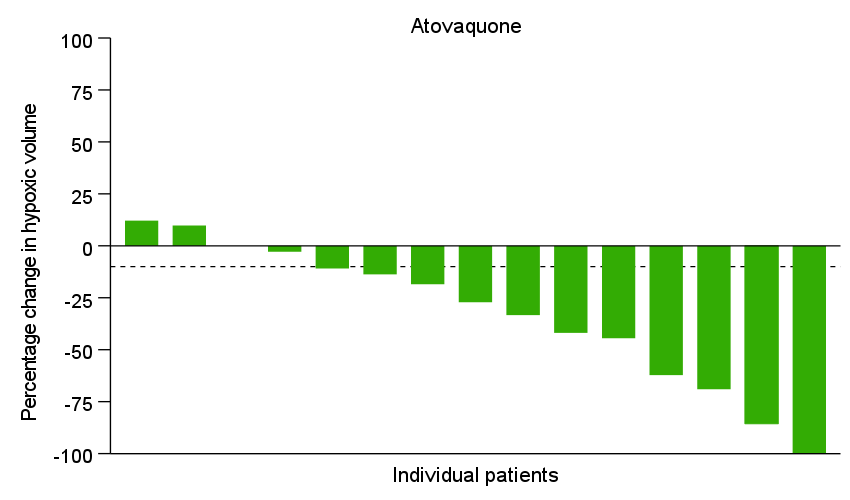

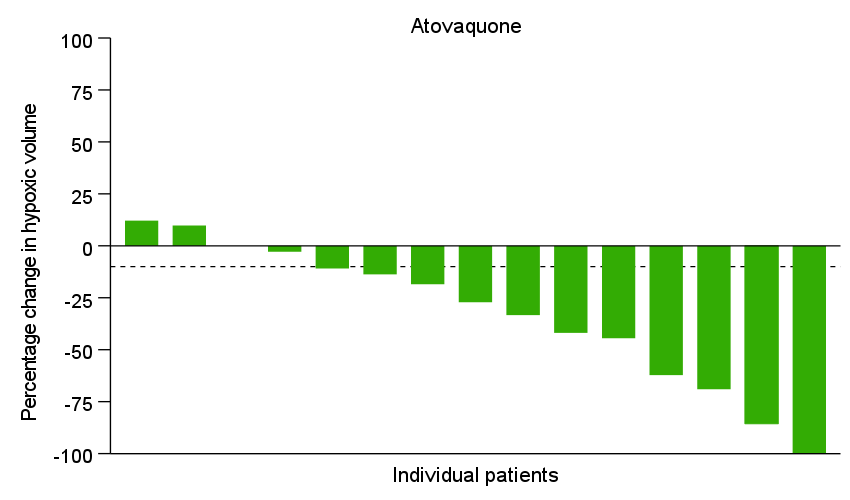

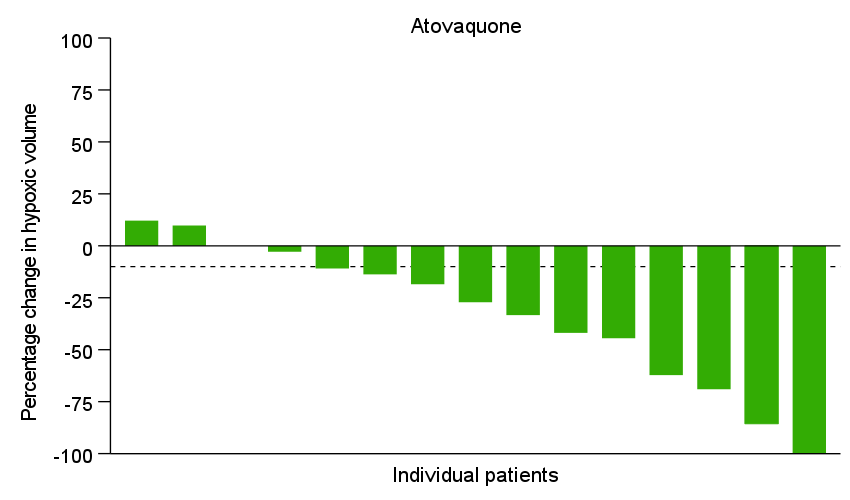

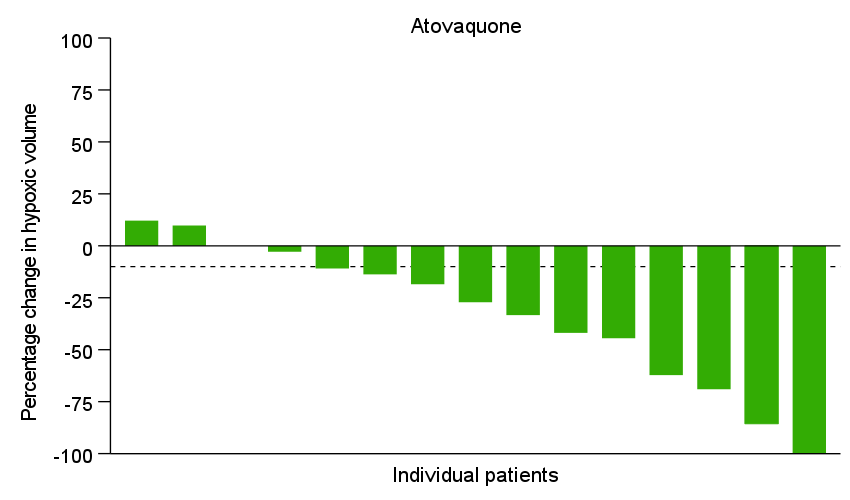

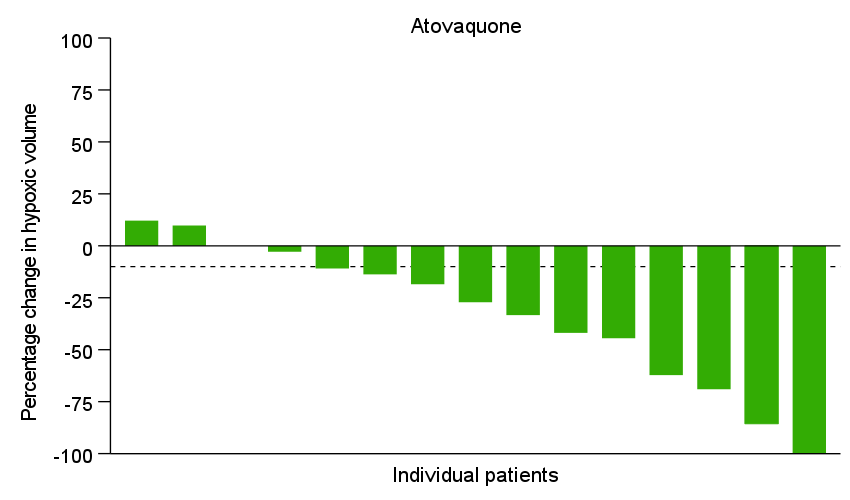

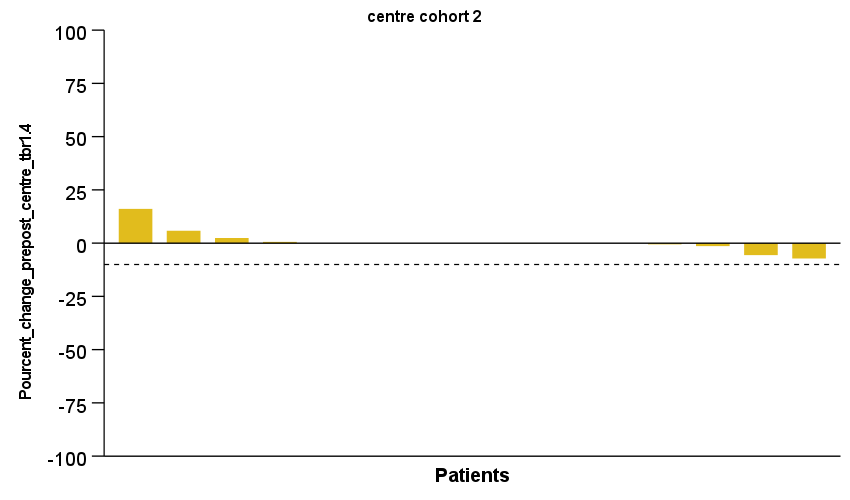

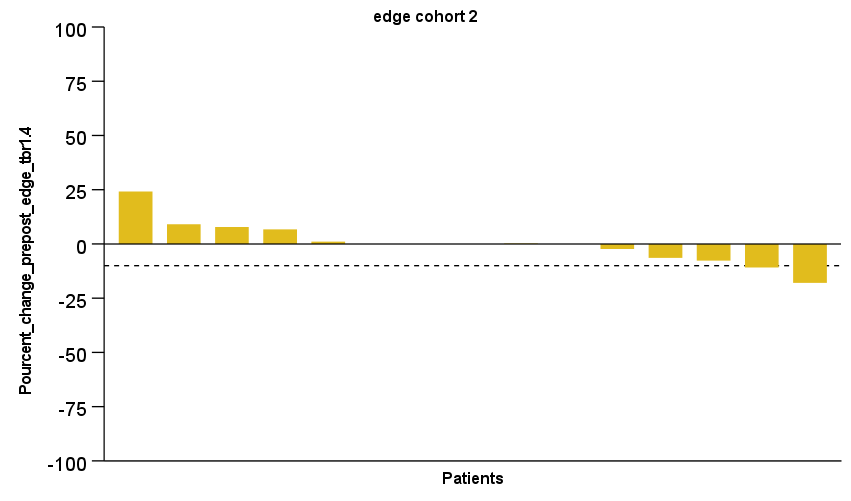


**Edge**

**Outer**

**D**

**C**

**A**

**Inner**

**Centre**

**B**

#### **Supplementary Figure 1.** Change in tumour HV measured by hypoxia PET-CT. Waterfall plots of percentage change in HV in tumour subregions for untreated patients: centre (**A**), inner (**B**), outer (**C**), and edge (**D**). TBR ≥ 1.4 is used to define HV. A reduction in HV ≥ 10% is considered meaningful. All untreated patients including those with no change in HV between baseline scan and pre-surgery scan are included.


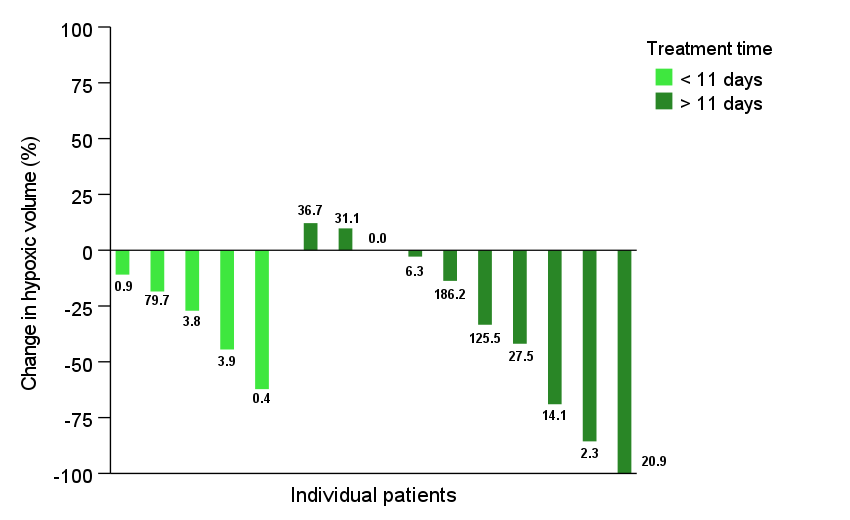

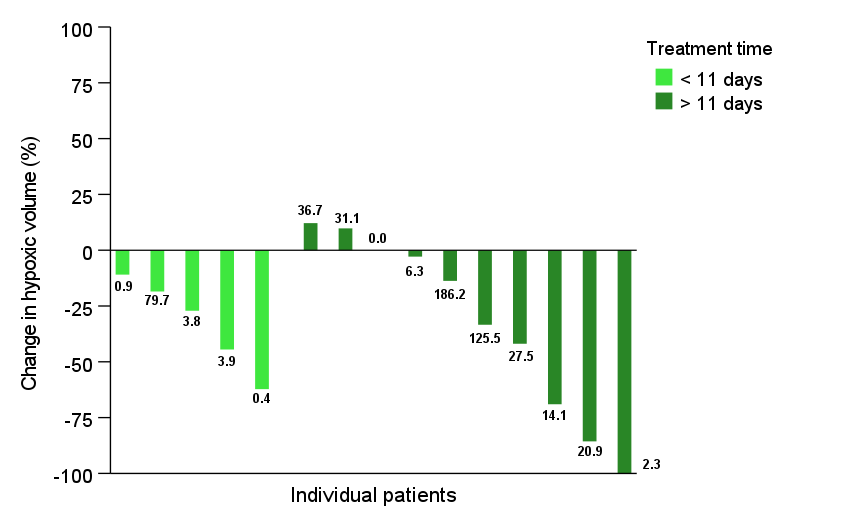


#### **Supplementary Figure 2.** Influence of treatment time on tumour hypoxic volume changes. Percentage change in tumour hypoxic volume in patients who were treated with atovaquone for less than 11 days (light green) or more than 11 days (dark green). The baseline tumour hypoxic volume (mL) is indicated for each patient. Hypoxic volume is defined by TBR ≥ 1.4.


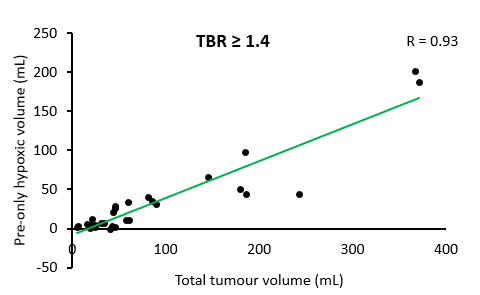


A


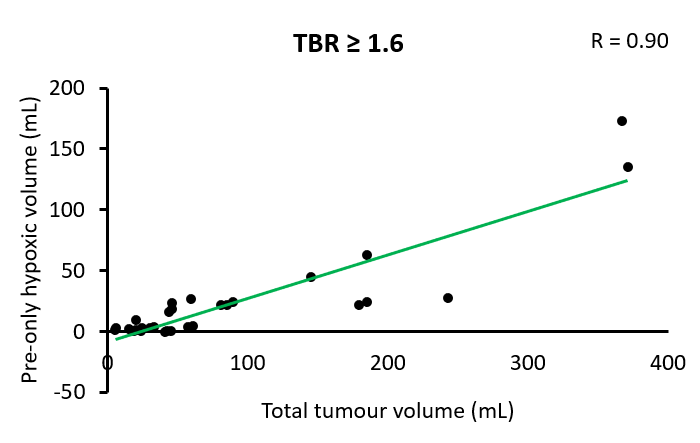


B


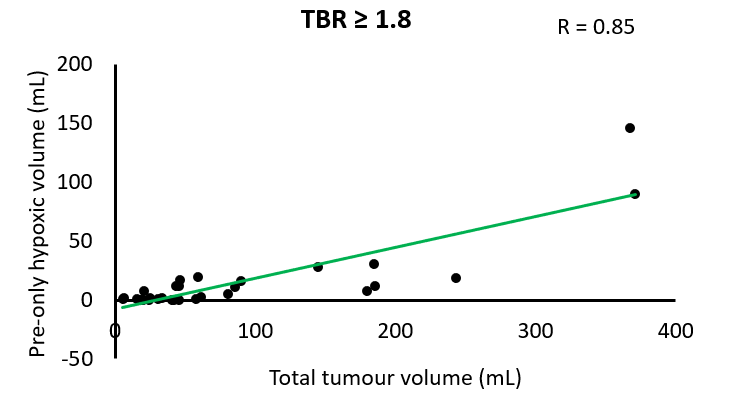


C

**Supplementary Figure 3.** Relationship between baseline tumour hypoxic volume and total baseline tumour volume for all patients (n = 30). Hypoxic volume is defined by TBR ≥ 1.4 (**A**), TBR ≥ 1.6 (**B**), or TBR ≥ 1.8 (**C**).
